# Structure and Protein–Protein Interactions of Human UDP-Glucuronosyltransferases

**DOI:** 10.3389/fphar.2016.00388

**Published:** 2016-10-24

**Authors:** Ryoichi Fujiwara, Tsuyoshi Yokoi, Miki Nakajima

**Affiliations:** ^1^Department of Pharmaceutics, School of Pharmacy, Kitasato UniversityTokyo, Japan; ^2^Department of Drug Safety Sciences, Division of Clinical Pharmacology, Nagoya University Graduate School of MedicineNagoya, Japan; ^3^Drug Metabolism and Toxicology, Faculty of Pharmaceutical Sciences, Kanazawa UniversityKanazawa, Japan

**Keywords:** UDP-glucuronosyltransferase (UGT), protein–protein interactions, glucuronidation, glucuronides, drug-metabolizing enzymes

## Abstract

Mammalian UDP-glucuronosyltransferases (UGTs) catalyze the transfer of glucuronic acid from UDP-glucuronic acid to various xenobiotics and endobiotics. Since UGTs comprise rate-limiting enzymes for metabolism of various compounds, co-administration of UGT-inhibiting drugs and genetic deficiency of *UGT* genes can cause an increased blood concentration of these compounds. During the last few decades, extensive efforts have been made to advance the understanding of gene structure, function, substrate specificity, and inhibition/induction properties of UGTs. However, molecular mechanisms and physiological importance of the oligomerization and protein–protein interactions of UGTs are still largely unknown. While three-dimensional structures of human UGTs can be useful to reveal the details of oligomerization and protein–protein interactions of UGTs, little is known about the protein structures of human UGTs due to the difficulty in solving crystal structures of membrane-bound proteins. Meanwhile, soluble forms of plant and bacterial UGTs as well as a partial domain of human UGT2B7 have been crystallized and enabled us to predict three-dimensional structures of human UGTs using a homology-modeling technique. The homology-modeled structures of human UGTs do not only provide the detailed information about substrate binding or substrate specificity in human UGTs, but also contribute with unique knowledge on oligomerization and protein–protein interactions of UGTs. Furthermore, various *in vitro* approaches indicate that UGT-mediated glucuronidation is involved in cell death, apoptosis, and oxidative stress as well. In the present review article, recent understandings of UGT protein structures as well as physiological importance of the oligomerization and protein–protein interactions of human UGTs are discussed.

## Introduction

Humans are exposed on a daily basis to xenobiotics that may potentially be toxic or pharmacologically active. Xenobiotics are often hydrophobic and therefore may accumulate in the body. To facilitate the excretion of xenobiotics, detoxifying enzymes metabolize them mostly in the liver to increase their hydrophilicity. Since such xenobiotic-metabolizing enzymes play an important role in the metabolism of clinically used drugs, they are also called drug-metabolizing enzymes. Phase I drug-metabolizing enzymes, such as cytochrome P450s (CYPs) and esterases, catalyze oxidation, reduction, and hydrolysis of xenobiotics (Satoh and Hosokawa, 1998; Nebert and Russell, 2002). The formed metabolites, as well as parental compounds, are further metabolized by phase II drug-metabolizing enzymes, such as UDP-glucuronosyltransferases (UGTs), sulfotransferases, and glutathione *S*-transferases (Jancova et al., 2010; Miners et al., 2010). Among these, UGTs have been reported with the highest contribution to drug metabolism (Williams et al., 2004).

UDP-glucuronosyltransferases-mediated glucuronidation can be a rate-limiting step in the clearance of endogenous and exogenous substances. Therefore, inhibition of UGTs by co-administered drugs or genetic deficiency in the *UGT* gene can increase blood concentrations of their substrates *in vivo*, whereas induction of UGT genes would result in a decrease of blood concentrations of their substrates (Lankisch et al., 2009; Hirashima et al., 2016). Three-dimensional crystal structures are useful to facilitate the understanding of protein structures that determine substrate- and/or inhibitor-binding. Due to the difficulty in obtaining the crystal structure of membrane proteins, entire three-dimensional structures of human UGTs have not been determined except for a partial domain of human UGT (Miley et al., 2007). On the other hand, entire crystal structures of soluble forms of plant and bacterial UGTs have previously been reported (Mulichak et al., 2003; Shi et al., 2014). These solved structures were used as templates of homology modeling of human UGTs (Fujiwara et al., 2009a). Although amino acid similarities are not high between mammalian and plant/bacterial UGTs, the modeled human UGT structures are comparable to plant and bacterial UGTs, suggesting that the structures of plant/bacterial UGTs may aid in the structural clarification of human UGTs.

UDP-glucuronosyltransferases enzymes comprise a superfamily. One of the most unique and important properties of UGTs is that they form homo- and hetero-oligomers such as dimers, trimers, and tetramers (Finel and Kurkela, 2008). Tukey and Tephly (1981) were the first to report that rat UGTs functioned in an oligomeric form. Subsequently, various *in vitro* techniques such as cross-linking and fluorescence resonance energy transfer (FRET) imaging demonstrated the oligomerization of UGT proteins (Ikushiro et al., 1997; Operaña and Tukey, 2007). Interestingly, accumulating evidence indicates that UGT–UGT interactions affect their enzymatic activities (Ishii et al., 2001; Fujiwara et al., 2007a,b). Analyses using the homology-modeled UGT structures further revealed the region responsible for the oligomerization of UGTs (Lewis et al., 2011). Moreover, specific antibodies against UGTs immunoprecipitated not only UGTs but also CYPs in human liver microsomes, indicating that UGTs appeared to interact with other microsomal proteins (Fujiwara and Itoh, 2014). Indeed, recently developed techniques such as mass spectrometry analysis of immunoprecipitates revealed that UGTs may interact with a variety of microsomal proteins including epoxide hydrolase 1, carboxylesterase 1, alcohol dehydrogenases, and glutathione *S*-transferases (Fujiwara and Itoh, 2014).

In this review article, recent advances in the knowledge on the three-dimensional structure, protein interactions of human UGTs, and physiological roles of UGTs are introduced along with early and recent analytical tools that demonstrate the presence of UGT oligomers.

## UDP-Glucuronosyltransferase (UGT)

### Human UGT Families and Their Function

**FIGURE 1:**
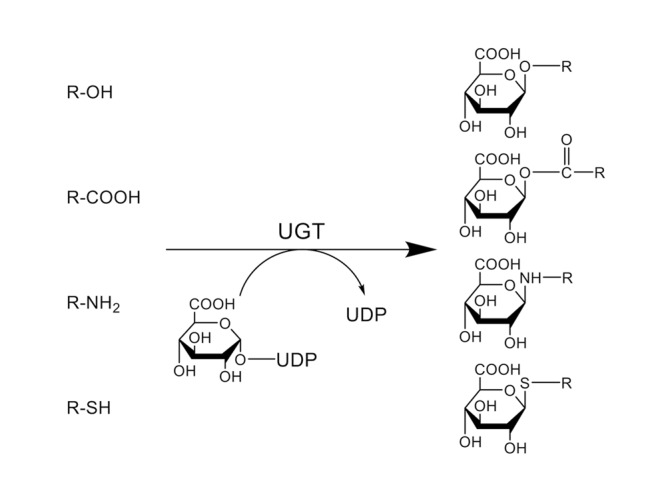
**Conjugation reaction catalyzed by UDP-glucuronosyltransferases (UGTs).** UGTs catalyze the transfer of glucuronic acid from UDP-glucuronic acid to an oxygen, nitrogen, or sulfur atom of their substrates. (R = substrate).

UDP-Glucuronosyltransferase (UGT, EC 2.4.1.17), which belongs to a large glycosyltransferase (GT) 1 family of GTs (EC 2.4.1.-; GT), is a family of membrane-bound proteins that catalyze a transfer of glucuronic acid from UDP-glucuronic acid to various endogenous and exogenous substances (**Figure 1**) (Mackenzie et al., 2005). UGTs are specifically expressed in the ER and most the UGTs are localized in the luminal side of the ER-membrane, which is rich in UDP-glucuronic acid. In humans, the *UGT* gene superfamily contains *UGT1* and *UGT2*.

**FIGURE 2:**
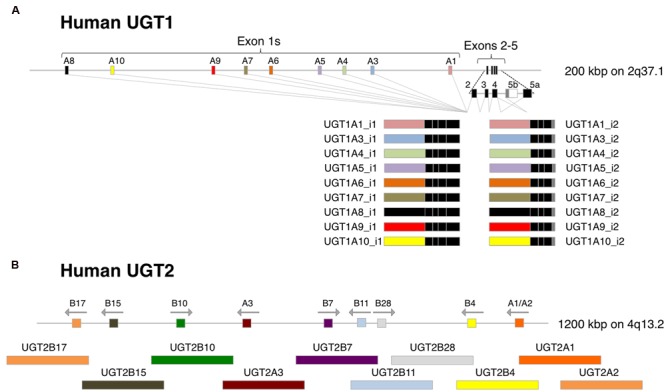
**Gene structures of human *UGT1* and *UGT2*. (A)** Human *UGT1* gene contains multiple exon 1s and common exons 2–5, and each UGT1 isoform are generated by exon sharing of the gene. Exon 5a produces UGT1A_i1 proteins, while exon 5b produced smaller UGT1A_i2 proteins. **(B)** UGT2A1 and UGT2A2 are generated by exon sharing of unique exon 1s and common exons 2–6 of the *UGT2A* gene in the same manner as UGT1A proteins. UGT2A3 and UGT2B family proteins are encoded by each unique gene in a cluster.

The single human *UGT1* gene, located on chromosome 2q37.1, contains multiple exon 1s and common exons 2–5, spanning approximately 200 kbp. Individual UGT1 isoforms, UGT1A1, UGT1A3, UGT1A4, UGT1A5, UGT1A6, UGT1A7, UGT1A8, UGT1A9, and UGT1A10, are generated by exon sharing of the *UGT1* gene (**Figure 2A**). Importantly, Dr. Girard et al. (2007) discovered that there are two types of exon 5, exons 5a and 5b, which encodes a shorter amino acid sequence. Compared to 50–55 kDa proteins encoded by exons 1–4 and 5a (UGT1A_i1), which is a main variant, the proteins encoded by exons 1–4 and 5b (UGT1A_i2) are smaller (45 kDa) and generally exhibit lower enzymatic activities.

Human *UGT2* genes, including *UGT2A* and *UGT2B*, are located on chromosome 4q13.2. UGT2A1 and UGT2A2 are generated by exon sharing of unique exon 1s and common exons 2–6 of the *UGT2A* gene in the same manner as UGT1A proteins, whereas a single gene encodes UGT2A3. UGT2B family proteins, UGT2B4, UGT2B7, UGT2B10, UGT2B11, UGT2B15, UGT2B17, and UGT2B28, are encoded by each unique gene in a cluster (**Figure 2B**). Transcriptional diversity has been reported in the *UGT2B7* locus. Original six exons as well as extra three exon 1s and two exon 6s of the *UGT2B7* gene can produce up to 22 transcript variants which encode 7 types of UGT2B7 proteins (UGT2B7_i1 to _i7) (Ménard et al., 2011). Similar to UGT1A_i1, UGT2B7_i1 exhibits the highest enzyme activity compared to UGT2B7_i2 to _i7 proteins. Recently conducted targeted RNA next-generation sequencing revealed that transcriptional diversity, such as new internal exons and exon skipping, could be observed in other *UGT2B* genes (Tourancheau et al., 2016). The expression and enzyme activities of such alternative UGT2Bs need to be determined in the future.

### Tissue Distribution of UGTs

In humans, all of 9 UGT1 and 10 UGT2 isoforms are expressed in a tissue-specific manner. In the liver, which is the most important tissue in metabolism of xenobiotics, UGT1A1, UGT1A3, UGT1A4, UGT1A6, UGT1A9, UGT2B4, UGT2B7, UGT2B10, UGT2B15, and UGT2B17 are expressed (Nakamura et al., 2008; Izukawa et al., 2009). UGT1A8 and UGT1A10 are mainly expressed in the small intestine, colon, and bladder. UGT1A7 has been characterized as an isoform that is specifically expressed in the stomach (Strassburg et al., 1997). In the kidneys, UGT1A9 and UGT2B7 are highly and other UGTs such as UGT1A4, UGT1A6, and UGT2B11 are moderately expressed. The expression of UGT2B28 is limited to the bladder, where various UGT1 and UGT2 members are also expressed. UGT2A1 and UGT2A2 are expressed in nasal tissue, whereas UGT2A3 is expressed mainly in liver and small intestine, and slightly in lung and nasal tissues (Sneitz et al., 2009). Since UGT2A family isoforms glucuronidate endogenous substances rather than drugs, it has been believed that they have certain physiological role in those organs, although more investigation is required.

### Substrate Specificity of UGTs

UDP-glucuronosyltransferases catalyze the transfer of glucuronic acid from UDP-glucuronic acid to an oxygen, nitrogen, or sulfur atom in their substrates (**Figure 1**). UGT1A1 glucuronidates relatively bulky molecules such as bilirubin, SN-38, and etoposide, as well as planar or smaller molecules such as estradiol, 1-naphthol, and 4-methylumbelliferone (Bosma et al., 1994; Watanabe et al., 2003; Mano et al., 2004). While these smaller compounds can be glucuronidated by several other UGT1 and UGT2 family proteins, bilirubin is solely glucuronidated by UGT1A1 (Bosma et al., 1994). UGT1A4 has been recognized as one of the UGT isoforms that can glucuronidate tertiary amines (e.g., nicotine, imipramine, trifluoperazine, and lamotrigine) (Kuehl and Murphy, 2003; Smith et al., 2003; Rowland et al., 2006). A recent study demonstrated that clearances of nicotine, amitriptyline, imipramine, and diphenhydramine by UGT2B10-mediated *N*-glucuronidation were significantly higher than those by UGT1A3 and UGT1A4, indicating that UGT2B10 plays an important role in *N*-glucuronidation of certain amine-containing compounds (Kato et al., 2013). As UGT1A6 was originally characterized as a phenol-glucuronidating enzyme, it mainly glucuronidates small phenolic substances such as 4-nitrophenol, 1-naphthol, and 4-methylumbelliferone (Hanioka et al., 2001). 5-Hydroxytriptamine, also called serotonin, has been recognized as a specific substrate of UGT1A6 (Krishnaswamy et al., 2003). UGT1A9 metabolizes a wide variety of substances such as mycophenolic acid, scopoletin, and entacapone (Kurkela et al., 2003; Bernard and Guillemette, 2004). Propofol has been used as a selective substrate of UGT1A9 in the livers, although it can also be glucuronidated by UGT1A7, UGT1A8, and UGT1A10 in the gastrointestine (Court, 2005). UGT2B isoforms are especially important in the glucuronidation of endogenous compounds such as androsterone, testosterone, and dihydrotestosterone. Zidovudine and morphine are specifically metabolized by UGT2B7 (Barbier et al., 2000). It has been shown that UGT2B15 is the major enzyme responsible for sipoglitazar glucuronidation in humans, while multiple UGT1A and UGT2B members slightly glucuronidate sipoglitazar (Nishihara et al., 2013). UGT2B17, which has more than 95% homology with UGT2B15, can glucuronidate a wide variety of exogenous and endogenous compounds, including coumarins, anthraquinones, flavonoids, and androgens (Turgeon et al., 2003). Therefore, each UGT isoform exhibits broad but distinct substrate specificities.

### Significance of Extrahepatic UGTs

Because bilirubin is solely glucuronidated by UGT1A1, genetic deficiency in the *UGT1A1* gene can result in an onset of severe hyperbilirubinemia (>20 mg/dL serum bilirubin) in humans (Beutler et al., 1998). Knockout of *Ugt1a1* in mice causes very severe hyperbilirubinemia (>15 mg/dL serum bilirubin), which is lethal within 11 days of birth due to the development of kernicterus (Nguyen et al., 2008). Since UGT1A1 is highly expressed in the liver, it was previously commonly believed that hepatic UGT1A1 mainly contribute to the bilirubin glucuronidation. However, a recent study demonstrated that liver-specific knockout of the *Ugt1* gene, including *Ugt1a1*, resulted in a mild increase of serum bilirubin (2 mg/dL serum bilirubin; Chen et al., 2013). Furthermore, increased expressions of intestinal UGT1A1 lead to decreased serum bilirubin levels (from 12 to 2 mg/dL) in 14-day-old humanized *UGT1* mice, indicating that intestinal UGT1A1 also plays an important role in bilirubin metabolism (Fujiwara et al., 2010b, 2012). UGT1A1 expressed in the skin and brain might also be responsible for bilirubin metabolism in neonates although to a lesser extent (Sumida et al., 2013; Kutsuno et al., 2015). In addition to UGT1A1, substantial expression of UGT1A8 and UGT1A10 mRNA are also observed in the small intestine (Nakamura et al., 2008). Since recombinant UGT1A1, UGT1A8, and UGT1A10 glucuronidate raloxifene *in vitro*, these enzymes might be responsible for the extremely poor oral bioavailability of raloxifene (Mizuma, 2009). However, it should be noted that the UGT1A8 protein was barely detected in human small intestine in a targeted peptide-based quantification study (Sato et al., 2014). Therefore, the role of UGT1A8 in intestinal glucuronidation *in vivo* needs to be carefully investigated in the future. These data support a concept that in addition to hepatic UGTs, extrahepatic UGTs also play a dominant role in glucuronidation of endogenous and exogenous compounds (Fujiwara et al., 2015).

## Three-Dimensional Structure of Human UGTs

### Structure of Glycosyltransferase 1 Family Protein

Due to difficulties in crystallizing membrane-bound protein, X-ray crystal structures of membrane-bound human UGTs have not been determined. As mentioned above, human UGTs belong to a large glycosyltransferase (GT) 1 family. Because plant and bacterial GT1 family proteins are soluble forms, several X-ray crystal structures of plant and bacterial GTs have successfully been determined. In 2003, a 2.8-Å crystal structure of TDP-*epi*-vancosaminyltransferase (GtfA), which is one of the GT1 family proteins that transfer 4-*epi*-vancosamine from TDP-*epi*-vancosamine to its substrate, in *Amycolatopsis orientalis* was solved (Mulichak et al., 2003). The crystal structure (PDB ID: 1PN3) revealed that GtfA adopts a GT-B fold that consists of two separate Rossmann domains with a connecting linker and a catalytic cleft. In addition, other GT1 family proteins such as plant UDP-glucose flavanoid 3-*O* glucosyltransferase (2C1X) and multifunctional triterpene/flavonoid glycosyltransferase UGT71G1 (2ACV) also have the GT-B fold (Shao et al., 2005; Offen et al., 2006). These findings lead us to hypothesize that human UGTs would also be GT-B fold enzymes.

### Predicted Structure of Human UGTs

Human UGT1 and UGT2 family members consist of approximately 530 amino acids. One of the unique properties of UGTs is that they sometimes recognize overlapping but specific substrates, while they commonly recognize a co-substrate UDP-glucuronic acid (Mackenzie et al., 2005). Because UGT1 family proteins are generated by exon sharing of the single *UGT1* gene (**Figure 2A**), their C-terminal amino acid sequences encoded by the common exons 2–5 are identical. Although UGT2 family proteins are encoded by their individual genes (**Figure 2B**), the C-terminal amino acid sequences exhibit extremely high amino acid similarity. In contrast, the N-terminal amino acid sequences of UGT1 and UGT2 family members exhibit relatively lower amino acid similarity. For example, the sequence homology is 24–49% between N-terminal regions of UGT1 family enzymes (Mackenzie et al., 1997). Point mutation analyses demonstrated that mutations in the N- and C-terminal halves dramatically decreased the affinities toward substrates and UDP-glucuronic acid, respectively (Xiong et al., 2006; Patana et al., 2007; Fujiwara et al., 2009b; Kerdpin et al., 2009). These mutagenesis studies support an assumption that human UGTs have two domains, the highly conserved C-terminal halves responsible for the UDP-glucuronic acid binding and the unique N-terminal halves responsible for the substrate binding. A 1.8-Å resolution apo crystal structure of the C-terminal half of human UGT2B7 (2O6L), which is a solely available X-ray crystal structure of human UGTs, confirmed that the C-terminal domain contained a preserved nucleotide-sugar binding site (Miley et al., 2007).

### Homology-Modeled Structure of Human UGTs

**FIGURE 3:**
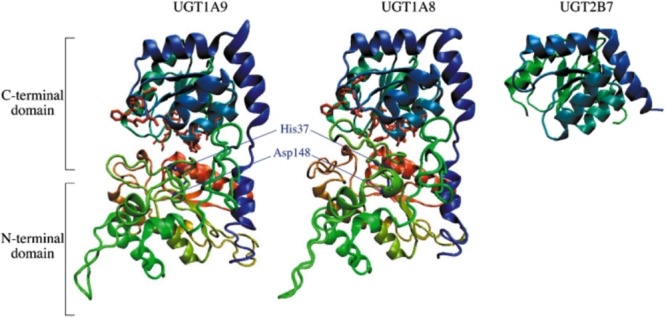
**Homology-modeled structures of human UGT1A9 and UGT1A8.** Three-dimensional structures of human UGT1A9 and UGT1A8 were homology-modeled using *Amycolatopsis orientalis* GtfA (PDB ID: 1PN3) as template structures. The structures of UGT1A9 and UGT1A8 contain two Rossmann fold-like domains in the N-terminus and C-terminus. The structures are colored from red at the N-terminal to blue at the C-terminal. Amino acid residues that are predicted to interact with UDPGA and to be important for the catalysis are shown by red and blue licorice, respectively. The X-ray crystal structure of the C-terminal domain of UGT2B7 was retrieved from the Protein Data Bank (PDB ID: 2O6L). Adapted from Drug Metabolism and Pharmacokinetics (Fujiwara et al., 2009a).

Three-dimensional structures of unsolved proteins can be modeled by homology-modeling. Locuson and Tracy (2007) conducted homology modeling of human UGT1A1 using the crystallized structure of plant UGT71G1 (2ACV) as a template. The length of the amino acid sequence of human UGT1A1 (533 amino acids) is relatively longer than that of plant UGT71G1 (463 amino acids). The amino acid similarity between UGT1A1 and UGT71G1 is 34%, but it was sufficient to obtain a reliable homology-model. The overall three-dimensional structure of UGT1A1 showed two Rossmann fold-like domains (Locuson and Tracy, 2007). Banerjee et al. (2008) modeled a three-dimensional structure of human UGT1A10 using UDP-galactose 4-epimerase (1XEL) from *Escherichia coli* as a template structure (Banerjee et al., 2008). The modeled structures of substrate- and UDP sugar-binding pockets were overlapped well with those of the template structure. The structural analysis indicated that lysine residues at 314 and 404 (K314 and K404) would play a critical role in the UDP-glucuronic acid binding of UGT1A10. They confirmed, by *in vitro* mutagenesis analysis, the importance of K314 and K404 in the UDP-glucuronic acid binding. Therefore, the homology-modeled structures of human UGTs are relatively reliable, even though the amino acid similarities are not high between human and plant/bacteria UGTs. Similar homology-modeling approaches have been carried out by many research groups to simulate the three dimensional structures of human UGTs (**Table 1**). Structural similarity of C-terminal domains was very high between the modeled structures and the crystallized structure of the C-terminal half of human UGT2B7 (2O6L), supporting the reliability of the homology-modeling technique (**Figure 3**).

**Table 1:** Homology modeled human UDP-glucuronosyltransferases (UGTs) and their template structures.

Modeled UGT	Template	Template PDB ID	Reference
UGT1A1	*Medicago truncatula* triterpene UDP-glucosyl transferase (UGT71G1)	2ACV	Locuson and Tracy, 2007
UGT1A1	*Medicago truncatula* triterpene UDP-glucosyl transferase (UGT71G1)	2ACV	Li and Wu, 2007
UGT1A1	*Arabidopsis thaliana* hydroquinone glucosyltransferase (UGT72B1)	2VCE	Laakkonen and Finel, 2010
	*Amycolatopsis orientalis* UDP-glycosyltransferase (GtfB)	1IIR	
	*Streptomyces antibioticus* oleandomycin glycosyltransferase	2IYA	
	*Homo sapiens* UGT2B7 (C-terminal domain)	2O6L	
UGT1A1	*Arabidopsis thaliana* hydroquinone glucosyltransferase (UGT72B1)	2VCE	Song et al., 2015
	*Streptomyces antibioticus* oleandomycin glycosyltransferase	2IYA	
	*Vitis vinifera* UDP-glucose flavonoid 3-*o*-glycosyltransferase (VvGTl)	2C1X	
UGT1A3	*Medicago truncatula* multifunctional (iso)flavonoid glycosyltransferase (UGT85H2)	2PQ6	Song et al., 2015
	*Vitis vinifera* UDP-glucose flavonoid 3-*o*-glycosyltransferase (VvGTl)	2C1X	
	*Medicago truncatula* Flavonoid 3-*o*-glucosyltransferase (UGT78G1)	3HBF	
UGT1A3	*Streptomyces antibioticus* oleandomycin glycosyltransferase	2IYA	Yao et al., 2015; Liu X. et al., 2016
	*Vitis vinifera* UDP-glucose flavonoid 3-*o* glycosyltransferase (VvGTl)	2C1X	
	*Arabidopsis thaliana* hydroquinone glucosyltransferase (UGT72B1)	2VCE	
UGT1A3	*Vitis vinifera* UDP-glucose flavonoid 3-*o* glycosyltransferase (VvGTl)	2C1Z	Schirris et al., 2015
UGT1A8 and UGT1A9	*Amycolatopsis orientalis* TDP-epi-vancosaminyltransferase (GtfA)	1PN3	Fujiwara et al., 2009a
UGT1A9	*Vitis vinifera* UDP-glucose flavonoid 3-*o-*glycosyltransferase (VvGTl)	2C1Z	Wu et al., 2012
UGT1A9	*Vitis vinifera* UDP-glucose flavonoid 3-*o*-glycosyltransferase (VvGTl)	2C1Z	Li et al., 2016
	*Homo sapiens* UGT2B7 (C-terminal domain)	2O6L	
UGT1A9 and UGT1A10	*Streptomyces antibioticus* oleandomycin glycosyltransferase	2IYA	Tripathi et al., 2016
	*Streptomyces fradiae* C-glycosyltransferase (UrdGT2)	2P6P	
	*Medicago truncatula* triterpene UDP-glucosyl transferase (UGT71G1)	2ACV	
	*Homo sapiens* UGT2B7 (C-terminal domain)	2O6L	
UGT1A10	*Escherichia coli* UDP-galactose 4-epimerase	1XEL	Banerjee et al., 2008
UGT2B7	*Vitis vinifera* UDP-glucose flavonoid 3-*o-*glycosyltransferase (VvGTl)	2C1X, 2C1Z, 2C9Z	Lewis et al., 2011; Chau et al., 2014
	*Medicago truncatula* triterpene UDP-glucosyl transferase (UGT71G1)	2ACV, 2ACW	
	*Homo sapiens* UGT2B7 (C-terminal domain)	2O6L	
UGT2B7	*Streptomyces antibioticus* oleandomycin glycosyltransferase	2IYA	Zhang et al., 2016
	*Medicago truncatula* multifunctional (iso)flavonoid glycosyltransferase (UGT85H2)	2PQ6	
C-terminal domains of UGT1, UGT2A1/2A2, UGT2A3, UGT2B4, UGT2B10, UGT2B11, UGT2B15, UGT2B17, UGT2B28	*Homo sapiens* UGT2B7 (C-terminal domain)	2O6L	Nair et al., 2015

Among the 19 functional human UGT proteins, UGT1A9 exhibits several unique properties. In 2007, we demonstrated that UGT1A9 was uniquely stable against heat treatment, while the other human UGTs lost their enzymatic activities when incubated at higher temperature (Fujiwara et al., 2007b). Importantly, 13 amino acid residues were found to be specific to UGT1A9 among 9 UGT1A isoforms. To examine the role of these residues in the thermal stability of UGT1A9, we conducted molecular dynamics simulation of homology-modeled structures of UGT1A9 as well as UGT1A8 as a reference at higher and lower temperatures. The *in silico* simulation revealed that the UGT1A9-specific residues were collectively involved in the thermal stability of UGT1A9 (Fujiwara et al., 2009b). *In vitro* mutagenesis analysis confirmed that the UGT1A9-specific residues, Arg42, Lys91, Ala92, Tyr106, Gly111, Tyr113, Asp115, Asn152, Leu173, Leu219, His221, Arg222, and Glu241, contributed to protein stability. Since the results of *in silico* and *in vitro* analyses were consistent, it is considered that the homology-modeled structure of human UGT1A9 (**Figure 3**) was relatively reliable.

## Dimerization and Oligomerization of UGTs

The first evidence to demonstrate the oligomerization of mammalian UGTs was reported by Tukey and Tephly (1981). Shortly after the publication, Matern et al. (1982) demonstrated oligomeric UGTs in. Subsequently, a number of research groups showed that mammalian UGTs including human UGTs formed homo- and hetero-oligomers such as dimers, trimers, and tetramers. In this section, earlier and recent analytical tools used to show the oligomeric UGTs are summarized.

### Gel Permeation Chromatography

Tukey and Tephly (1981) purified two different UGT isoforms, which mediate estrone and *p*-nitrophenol glucuronidations, respectively, from rabbit liver microsomes by DEAE-cellulose chromatography and affinity chromatography on UDP-hexanolamine Sepharose-4B. Both enzymes exhibited molecular weights of 57 kDa in the SDS-PAGE analysis. Interestingly, a gel filtration study of the purified UGT isoforms by an Ultragel AcA 34 column revealed that both UGTs had apparent molecular weights of 230 kDa (Tukey and Tephly, 1981), which is approximately 4 times larger than the size of monomeric UGTs. This was the first report demonstrating that mammalian UGTs could be present as tetramers.

Matern et al. (1982) similarly purified UGTs that catalyze chenodeoxycholic acid and testosterone glucuronidations from rat liver microsomes by a series of purification steps such as polyethylene glycol fractionation, DEAE-Sepharose CL-6B chromatography, UDP-hexanolamine-Sepharose 4B chromatography, and Bio-Gel A-1.5 m chromatography. While the molecular weight of subunit was determined to be 54 kDa in the SDS-PAGE analysis, the apparent molecular weight of the enzyme was calculated to be 316 kDa in a polyacrylamide gradient slab gel electrophoresis (Matern et al., 1982). The data indicated that rat UGTs were also present as tetrameric or even larger oligomeric forms.

### Radiation Inactivation

Radiation inactivation is an analytical tool to determine molecular weights of membrane-bound enzymes *in situ* (Kempner and Schlegel, 1979). Enzymes are inactivated when they are irradiated with ionizing radiation. The extent of radiation-induced inactivation of the enzymes is directly associated with the radiation dose and the molecular weight of the concerned enzymes. Peters et al. (1984) determined the molecular weights of rat UGTs by radiation-inactivation of SDS-treated lyophilized liver microsomes using a calibrated ^60^Co source. The radiation-inactivation analysis revealed that bilirubin mono-glucuronidation was catalyzed by a 41.5 kDa protein, while bilirubin di-glucuronidation was catalyzed by a 175 kDa protein. It was further demonstrated that proteins with molecular weight of 142 and 159 kDa catalyzed glucuronidations of testosterone and phenolphthalein, respectively (Peters et al., 1984). Similarly, the radiation inactivation analysis by Gschaidmeier and Bock (1994) revealed that molecular weights of UGTs catalyzing the glucuronidations of 1-naphthol, 6-hydroxychrysene, 3,6-dihydroxybenzo[*a*]pyrene, and 3,6-dihydroxychrysene were 91–218 kDa. The series of radiation inactivation analyses clearly demonstrated that mammalian UGTs were functional as oligomeric proteins that are composed of two to four subunits.

### Cross-Linking

In intact cells, most of proteins are interacting with other protein(s) (Jeong et al., 2001). Upon the denaturing process in the SDS-PAGE analysis, such protein interactions via disulfide binding, hydrogen binding, and hydrophobic or salt-bridge interactions are disrupted, so that the proteins are separated according to their molecular weight. Cross-linkers are reagents that can introduce covalent chemical bonds between specific amino acids of proteins. Thus, proteins treated with cross-linkers can be observed as bands with higher molecular weights rather than their monomeric forms on SDS-PAGE followed by immunoblotting. Ikushiro et al. (1997) used 1,6-bis(maleimido)hexane (BMH), which is a homobifunctional cross-linker that reacts with sulfhydryl-groups of proteins. When rat liver microsomes that were incubated with BMH and were applied to the SDS-PAGE followed by the immunoblotting analysis using anti UGT1 or UGT2B1 antibodies, multiple bands with molecular masses of 50–60 kDa as well as of 120–130 kDa were observed (Ikushiro et al., 1997). They concluded that sulfhydryl group(s) of rat UGTs are located on the outside of the proteins and play a critical role in the formation of UGT dimers. Ghosh et al. (2001) used the disulfide cross-linker BMH and an amino group cross-linker dimethyl 3,3′-dithiobispropionimidate (DTBP) to demonstrate homo-oligomers of human UGT1A1, finding that both BMH and DTBT produced bands corresponding to homo-dimers of UGT1A1 on the SDS-PAGE analysis. The density of the bands was apparently reduced when UGT1A1-expressed cells were incubated with DTBP at pH 9.0, indicating that homodimerization of human UGT1A1 can be disrupted at alkaline pH.

### Affinity Purification and Immunoprecipitations

Immunopurification and immunoprecipitation are classical methods to identify proteins interacting with a target protein. When rat liver microsomes were solubilized in buffer containing 1% Emulgen 913 and were applied to a UGT1 antibody-conjugated Sepharose 4B column, not only UGT1 isozymes but also unidentified 50-kDa protein(s) were co-eluted by eluting solution containing UGT1A-peptides (Ikushiro et al., 1997). Amino acid sequencing of the 50-kDa proteins and immunoblotting studies further revealed that the protein interacting with UGT1A was UGT2B1. Although the method used in a study by Kurkela et al. (2003) was Ni-column which is not immunopurification, they purified homo-oligomer of UGT1A9 that consisted of His- and hemagglutinin (HA)-tagged UGT1A9.

Fremont et al. (2005) conducted immunoprecipitation assays to examine protein–protein interactions between UGT1A1, UGT1A6, and UGT2B7 in human liver microsomes. When solubilized microsomes were incubated with UGT1A1-, UGT1A6-, and UGT2B7-specific antibodies, not only the antigenic proteins but also other UGT isozymes were co-immunoprecipitated. In 2007, we established cell lines that are individually or simultaneously expressing human UGT1A6 and UGT1A9 (Fujiwara et al., 2007b). A human UGT1A6-specific antibody co-immunoprecipitated UGT1A9, as well as UGT1A6, in the UGT1A6-UGT1A9 double expression cells, although it did not immunoprecipitate UGT1A9 in the UGT1A9-expressed cells. When cyan fluorescent protein (CFP)- and HA-tagged UGT1A1s were simultaneously expressed in COS cells, anti-HA beads immunoprecipitated both CFP- and HA-tagged UGT1A1s (Operaña and Tukey, 2007). Taken together, immunoaffinity purification and immunoprecipitation assays are typical but still powerful tools to demonstrate homo- and hetero-oligomerization of UGTs. Immunoprecipitation assays by Bellemare et al. (2010) further revealed that inactive UGT1A_i2 proteins form not only homo-oligomers (i2-i2) but also hetero-oligomers with UGT1A_i1 proteins (i1-i2).

### Fluorescence Resonance Energy Transfer (FRET)

To examine UGT–UGT interactions in intact cells, Operaña and Tukey (2007) conducted a FRET analysis using cyan and yellow fluorescent proteins (CFP and YFP)-tagged recombinant human UGTs expressed in COS cells. When correction for donor and acceptor bleed through was performed and FRET signal was analyzed, it was revealed that the two fusion UGT proteins resided within ångströms from each other. Their FRET analysis demonstrated homo-oligomerization of all UGT1A isoforms as well as hetero-oligomerization of UGT1A1 with the other UGT1A isoforms. The FRET analysis conducted by a different research group indicated that not only UGT1As, but also human UGT2B7 formed homo-oligomers in SF9 cells (Yuan et al., 2015; Liu Y.Q. et al., 2016).

## Regions and Amino Acid Residues Responsible for Oligomerization of UGTs

### Importance of N-terminal Domain

To examine the region responsible for the oligomerization of UGTs, Meech and Mackenzie (1997) constructed a couple of chimeric rat UGT2B1 proteins fused with ecdysteroid glucosyltransferase (EGT), which does not oligomerize. While intact UGT2B1 formed dimers, the dimerization was not observed when the C-terminal half of UGT2B1 was fused with the N-terminal half of EGT. In 2001, two-hybrid analysis by Ghosh et al. (2001) showed that UGT1A1s that were mutated or partially deleted in their N-terminal region (L175E, C233Y, or del152-180) abolished the ability to form homo-oligomers, while UGT1A1 with partial truncation of the C-terminal (K530X) still formed homo-oligomers (Ghosh et al., 2001). These data indicated that the N-terminal domains are involved in oligomerization of mammalian UGTs.

### Hydrophobic Amino Acids on the Surface of Modeled UGT2B7

Hydrophobic amino acid residues on the surface of proteins can mediate protein–protein interactions by introducing proline brackets and π–π interactions. To identify such hydrophobic amino acid residues of UGTs, Lewis et al. (2011) obtained a three-dimensional structure of human UGT2B7 by homology modeling (**Table 1**). In the homology-modeled structure of human UGT2B7, a cluster of highly hydrophobic amino acid residues on a B′-C loop (amino acid residues 183–200) was located on the protein surface (Lewis et al., 2011). Thus, not only *in vitro* studies, but *in silico* structural analyses also supported the involvement of the N-terminal region of UGTs in oligomerization.

## Protein–Protein Interactions of UGTs With Other Proteins

### Protein Interactions with CYPs

Cytochrome P450s are phase I drug-metabolizing enzymes that are expressed in the ER membrane. To investigate the possible protein-interactions between UGTs and CYPs, Taura et al. (2000) applied solubilized rat liver microsomes to a CYP1A1-conjugated Sepharose 4B column. It was found that multiple UGT isoforms were co-eluted in a fraction where CYP1A1 was eluted. Rat UGTs were detected in immunoprecipitates when solubilized rat liver microsomes were reacted with specific antibodies against CYP3A2, CYP2B2, CYP2C11, and CYP1A2 (Ishii et al., 2007). Antibodies against human UGT2B7 and CYP3A4 immunoprecipitated not only their antigenic proteins but also CYP3A4 and UGT2B7, respectively, in solubilized human liver microsomes (Fremont et al., 2005; Takeda et al., 2005, 2009). These data indicate that mammalian UGTs interact with CYPs in liver microsomes. It remains to be determined whether such UGT-CYP interactions can still be observed in a reconstituted system.

### Protein Interactions with Microsomal Proteins

Immunoprecipitation assay with human UGT2B7 antibody was conducted using solubilized human liver microsomes. The obtained immunoprecipitate was digested with trypsin, and the resulting peptides were analyzed by LC-MS/MS to identify proteins interacting with UGT2B7 in human liver microsomes (Fujiwara and Itoh, 2014). The extensive peptide analysis showed that the peptide sequences of UGT2B7, epoxide hydrolase 1, carboxylesterase 1, alcohol dehydrogenases, and glutathione *S*-transferases, as well as CYPs, were included in the immunoprecipitates. It was confirmed that such peptide sequences were not detected in immunoprecipitates obtained with a control rabbit IgG antibody. Therefore, UGT2B7 might be able to form a metabolosome, which is a functional unit of metabolism (Mori et al., 2011) in liver microsomes.

### Protein Interactions with Cytoplasmic Proteins

In contrast to normal UGT1A proteins (UGT1A_i1) that are expressed in the luminal side of the ER membrane, a portion of UGT1A_i2 is cytoplasmic (Lévesque et al., 2007). Immunoprecipitates of solubilized human intestine and kidney homogenates with anti-UGT1A_i2 antibody were applied to a global peptide analysis (Rouleau et al., 2014). It was found that cytoplasmic catalase and peroxiredoxin 1 were co-immunoprecipitated with UGT1A_i2 proteins in both tissues, indicating that the truncated UGT1A isoform 2 is interacting with those cytoplasmic proteins. Since such protein–protein interactions were not observed when anti-UGT1A_i1 antibody was used, the interactions with cytoplasmic proteins would be specific to UGT1A_i2 proteins.

## Physiological Significance of Oligomerization and Protein–Protein Interactions of UGTs

### Impact of UGT–UGT Interactions on the UGT-Mediated Glucuronidations

To investigate the effect of UGT–UGT interactions on the UGT activities, Ishii et al. (2001, 2004) cloned guinea pig UGT2B21 and UGT2B22 and examined morphine 6-*O*-glucuronidation in COS-7 cells expressing these UGTs. While UGT2B21 glucuronidates morphine, UGT2B22 does not have such ability to glucuronidate morphine. Morphine 6-glucuronide formation was 4.5-fold higher in COS-7 cells co-transfected with UGT2B21 and UGT2B22 compared to that in the cells transfected with UGT2B21 alone, indicating that protein–protein interactions between UGT2B21 and UGT2B22 upregulated the UGT activities (Ishii et al., 2001, 2004). This observation led us to investigate the impact of UGT–UGT interactions on the enzyme activities in humans (Fujiwara et al., 2007b; Nakajima et al., 2007). When we established stable expression systems of double human UGT1As in HEK293 cells, the *S*_50_ value of UGT1A1-mediated bilirubin glucuronidation was decreased by twofold by the co-expressions of UGT1A4 or UGT1A6 (Fujiwara et al., 2007a). A similar decrease of the *S*_50_ value was observed in the UGT1A1-mediated estradiol 3-*O*-glucuronidation in a co-expression system of UGT1A1 and UGT2B7 in HEK293 cells (Fujiwara et al., 2010a). These data showed that substrate-binding affinity of UGT1A1 toward bilirubin and estradiol was increased when UGT1A1 was co-expressed with UGT1A4, UGT1A6, and UGT2B7. Meanwhile, co-expression of UGT1A9 decreased the *V*_max_ value of UGT1A1-mediated estradiol 3-*O*-glucuronidation without affecting the *S*_50_ value (Fujiwara et al., 2007b). Kurkela et al. (2004) demonstrated that the rate of UGT1A9-catalyzed scopoletin glucuronidation was significantly decreased by co-expression of UGT1A4. Thus, UGT–UGT-interactions can modulate the catalytic rate of glucuronidation as well as the affinity of substrates toward UGTs. Importantly, the effects are dependent on interacting UGT isoforms and compounds used as substrates.

Interestingly, even though sorafenib is mainly metabolized by UGT1A9, it was demonstrated that the AUC of sorafenib was twice higher in patients with UGT1A1 variants or with hyperbilirubinemia (Peer et al., 2012). This finding indicates that UGT1A1 might control the enzyme activity of UGT1A9 *in vivo* by interacting with UGT1A9.

Although UGT1A_i2 proteins carry a potential UDP-glucuronic acid binding-site, they do not show substantial glucuronidation activity. As described above, the inactive UGT1A_i2 proteins form hetero-oligomers with functional UGT1A_i1 proteins. It was shown that glucuronide formation mediated by UGT1A_i1 proteins was significantly suppressed when UGT1A_i2 was co-expressed with UGT1A_i1 (Bellemare et al., 2010). The expression level and the ratio of UGT1A_i1 and _i2 are different in each tissue (Girard et al., 2007), suggesting that UGT1A_i2 can be a factor suppressing the glucuronidation in certain tissues. Interestingly, such suppression of UGT1 activity can also be caused by a truncated mutant of UGT1A1 (Gln331Stop; C to T at nucleotide 991), possibly by forming oligomers (Koiwai et al., 1996).

### Impact of UGT-CYP Interactions on the UGT Activities

While a *K*_m_ value of morphine 3-glucuronide formation was 0.38 mM in UGT2B7-expressing COS-1 cells, a much higher *K*_m_ value (3.7 mM) was observed in UGT2B7 and CYP3A4 co-expressing cells (Takeda et al., 2005). In contrast, co-expression of CYP1A2 or CYP2C9 did not affect the kinetic parameters of UGT2B7-catalyzed morphine 3-glucuronide formations. In addition, co-expression of CYP3A4 increased a *K*_m_ value of UGT1A6-mediated serotonin glucuronidation by ∼fourfold, whereas it barely affected *K*_m_ values of UGT1A1-mediated 4-MU, SN-38-, or estradiol 3-*O*-glucuronidations as well as UGT1A7-mediated 4-MU, SN-38-, and 4-hydroxybiphenyl-glucuronidations (Ishii et al., 2014). Meanwhile, co-expression of CYP3A4 increased *V*_max_ values of UGT1A1-mediated 4-MU, SN-38-, and estradiol 3-*O*-glucuronidations, UGT1A6-mediated serotonin glucuronidation, and UGT1A7-mediated 4-MU, SN-38-, and 4-hydroxybiphenyl-glucuronidations, although it did not affect the UGT2B7-mediated morphine 3-glucuronidation (Takeda et al., 2005; Ishii et al., 2014). Therefore, CYP isoforms differently affect UGT-mediated glucuronidations and the effects are depending on UGT isoforms as well as their substrates.

### Effect UGTs on Other Physiological Functions

Interestingly, a more than 40-fold interindividual variability in UGT1A6-catalyzed serotonin glucuronidation was observed among individual human liver microsomes (Krishnaswamy et al., 2003, 2005). Such variability could not be explained even when the activities were normalized with the UGT1A6 content and its genetic polymorphisms, indicating that there might be some unidentified factor that is capable of modulating the UGT1A6 activity. Since CYP isoforms can modulate UGT activities in different ways, interindividual variability in the CYP expression and function might be one of the causes of these wide interindividual variabilities in UGT1A6-catalyzed serotonin glucuronidation.

Miyauchi et al. (2015) investigated the effect of UGT2B7 on CYP3A4 activities by establishing a co-expression system of UGT2B7 and CYP3A4. They found that co-expression of UGT2B7 significantly decreased the *V*_max_ value of CYP3A4 activity without affecting a *K*_m_ value using luciferin-6′-pentafluorobenzyl ether as a substrate. Similarly, co-expression of UGT2B7 decreased the CYP3A4-mediated testosterone 6β-hydroxylation at a single substrate concentration (Miyauchi et al., 2015). These observations indicated that UGTs might suppress the enzyme activities of CYPs.

In a recent study by Liu M. et al. (2016), it was demonstrated that silencing of UGT1A expressions led to inhibitions of actinomycin D- and etoposide-induced p53 expressions in human colon HT29 and LS180 cells. Tissue-specific deletion of the *Ugt1* locus in intestinal crypt stem cells not only reduced p53 activation, but also compromised apoptosis. It was further demonstrated that reduced expression of UGT1A in intestine caused greater size and number of tumors in the colon cancer models (Liu M. et al., 2016).

When UGT1A_i2 was knocked-down in colorectal carcinoma-derived HT115 cells, expressions of a number of genes were up- or down-regulated (Rouleau et al., 2014). Among those genes, hemoglobin-alpha was significantly induced by the knockdown of UGT1A_i2. The function of hemoglobin-alpha proteins has been linked to cellular antioxidant potential (Widmer et al., 2010). Furthermore, it was found that knocked-down of UGT1A_i2 reduced a chemical-induced ROS formation in HT115 cells and significantly induced superoxide dismutase 1, an antioxidative gene. Although the underlying mechanism of induction of hemoglobin-alpha by knockdown of UGT1A_i2 is still unknown, the observation clearly indicates that UGT1A_i2 proteins are involved in the regulation of oxidative stress in cells. Taking all these findings together, it appears that UGTs are multifunctional proteins that controls metabolism, cell death and development of tumors, and oxidative stress.

## Critique and Future Research Directions

### Protein Structure of UGTs

Various three-dimensional structures of human UGTs have been simulated by homology modeling (**Table 1**) and they are relatively acceptable as mentioned above. However, due to the extremely low amino acid similarity and different length of the amino acid sequence between human UGTs and template plant and bacterial UGTs, the reliability of the modeled human UGT protein structures is still an open question. Indeed, it is generally believed that the identity of amino acid sequence with the template needs to be more than 70% to obtain reliable structure. Furthermore, there is a significant structural difference between human and plant/bacterial UGTs that human UGTs are membrane proteins while plant and bacterial UGTs are not. To obtain a completely reliable modeled structure of human UGTs, an X-ray crystal structure of human UGTs needs to be successfully solved. Importantly, as mammalian UGTs abolish their enzyme activities when they are solubilized, a solubilized and crystallized structure of human UGTs might be an enzymatically inactive form. The instability of the protein structure of human UGTs can also be an obstacle in crystallizing them. Interestingly, UGT1A9 solely exhibited its enzymatic activity in the presence of 0.2% Triton X-100, although activities of other UGT1A and 2B isoforms were abolished by the detergent treatment (Kurkela et al., 2003). Human UGT1A9 is uniquely stable against a heat treatment as well (Fujiwara et al., 2009a), indicating that the protein structure of UGT1A9 must be more stable than the structures of other UGT isoforms. Therefore, human UGT1A9 is a promising human UGT isoform that has a potential to be crystallized as an active form.

### Oligomerization of UGTs *In vivo*

**FIGURE 4:**
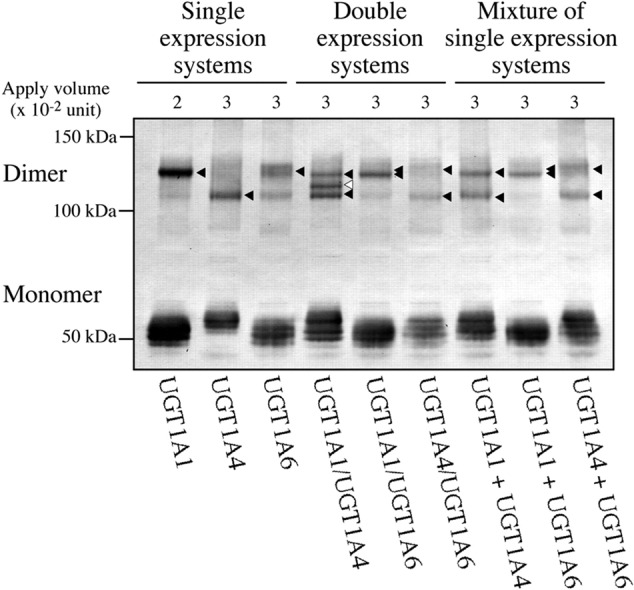
**Native PAGE analyses of UGT1A isoforms in single or double expression systems.** Single or multiple UGT1A isoforms were stably expressed in HEK293 cells. Total cell homogenates were solubilized with 1% Triton X-100 and 0.2% SDS, and then were subjected to a native gel. Immunoblotting was conducted with rabbit anti-human UGT1A antibody. The bands with closed and open arrowheads indicate homo- and hetero-dimers, respectively. Hetero-dimer was detected in the double expression system of UGT1A1 and UGT1A4, while such oligomer was not observed when single expression systems of UGT1A1 and UGT1A4 were mixed. Adapted from Drug Metabolism and Disposition (Fujiwara et al., 2007a).

Gel filtration and radiation inactivation analyses, where the liver microsomes were mixed with detergents, showed that monomeric UGTs catalyzed glucuronidation of certain compounds. It is well known that detergents can disrupt oligomerization of proteins; therefore, the monomeric UGTs might have been artificially generated from disruption of UGT oligomers. While SDS-PAGE shows the molecular weight of individual subunits of proteins under the reduced and denatured condition, oligomerization can be preserved in native-PAGE analysis. Our native-PAGE analysis of recombinant human UGTs expressed in HEK293 cells showed that most of UGTs were present as monomers, whereas some of them were still forming oligomers (**Figure 4**) (Fujiwara et al., 2007a). However, again, such monomers could have been a result from disruption of oligomeric UGTs due to a fact that the native-PAGE experiment required 1% Triton X-100 and 0.2% SDS to slightly solubilize the cell homogenates. Therefore, it is unclear whether UGTs dominantly exist as oligomer or monomer in intact cells.

Fluorescence resonance energy transfer is an analytical tool to demonstrate oligomerization of proteins in intact cells. Interestingly, FRET efficiency, which is a marker for co-localization and interaction, was 90% in COS cells expressing CFP- and YFP-tagged UGT1A7s (Operaña and Tukey, 2007), indicating that most of UGT1A7 was interacting with each other to form oligomers in intact cells. Meanwhile, the FRET efficiency was lower in certain UGT1A isoforms such as UGT1A3 and UGT1A9. These observations indicate that UGTs would exist as both monomeric and oligomeric forms in intact cells with variable extent depending on UGT isoforms. A limitation of the FRET analysis is that tagged UGT proteins, not intact proteins, were used. Therefore, determination of the extent of monomeric/oligomeric forms of intact UGT *in cellulo* is a future issue.

### Availability and Application of *Ugt1* and *Ugt2* Knockout Mice

Various data indicate that UGTs can be multifunctional proteins that are involved in cellular metabolism, apoptosis, carcinogenesis, and oxidative stress as well as glucuronidation. However, these unique properties of UGTs have not been fully validated by *in vivo* data. *Ugt1*- and *Ugt2*-knockout animals are promising *in vivo* models for investigating the roles of UGTs in such cellular metabolism, apoptosis, carcinogenesis, and oxidative stress. With extensive efforts by Drs. Tukey and Koller and their colleagues, *Ugt1*- and *Ugt2*-knockout mice were previously developed in 2008 and 2015, respectively (Nguyen et al., 2008; Fay et al., 2015). While *Ugt2* knockout mice can be used for the phenotype analysis, *Ugt1* knockout mice are lethal within 11 days after birth due to development of a bilirubin-induced irreversible brain damage, kernicterus. To conduct phenotype analyses in *Ugt1* knockout mice, therefore, we need to first avoid the lethality of the mice. Inhibitors of bilirubin production and inducers of bilirubin clearance might have potentials to rescue the lethality of the *Ugt1* knockout mice.

### Possible Role of UGTs in Transport of UGT Substrates and Metabolites

**FIGURE 5:**
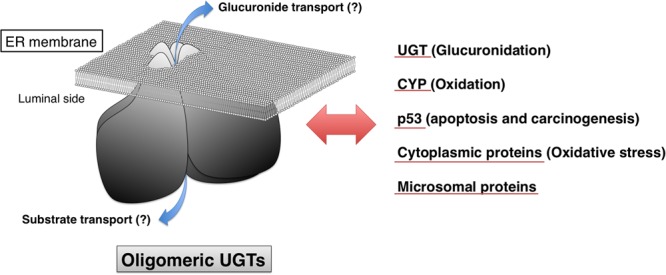
**Proposed model of oligomeric human UGTs.** Human UGTs form homo- and hetero-oligomers in ER membrane. As indicated with blue arrows, oligomerization of UGTs might be involved in the transport of substrates and glucuronides across the ER membrane. Protein–protein interactions of human UGT with other microsomal and cytoplasmic proteins such as UGT, CYP, and p53 can make a great impact on glucuronidation, oxidation, apoptosis, carcinogenesis, and oxidative stress.

Transporters responsible for the uptake of the co-substrate UDP-glucuronic acid from cytosol to the luminal side of ER membrane have been identified (Muraoka et al., 2001). Although studies suggested that there are proteins that are involved in the eﬄux of hydrophilic glucuronide across the ER membrane (Bánhegyi et al., 1996; Csala et al., 2004), such transporter(s) has not been identified. As shown in **Figure 5**, it was originally hypothesized that oligomerization and/or protein–protein interactions of UGTs have a role in transporting glucuronides across the ER membrane (Lin and Wong, 2002; Ishii et al., 2005); however, such evidence has not yet been obtained. Even in the immunoprecipitates of liver microsomes with anti-UGT2B7 antibody, no peptide sequences of transporters was observed (Fujiwara and Itoh, 2014). Further studies are required to fully understand the detailed information on the transport of UGT substrates and their metabolites across the ER membrane.

## Conclusion

In addition to enzyme inhibition and induction, oligomerization is a predominant factor regulating the function of UGTs. UGTs are multifunctional proteins that are involved not only in glucuronidation, but also in cellular metabolism, apoptosis, carcinogenesis, and oxidative stress in the human body (**Figure 5**). Global protein interactions of UGTs with other microsomal or cytoplasmic proteins might be a contributing factor to a wide interindividual variability in UGT-catalyzed glucuronidations.

## Author Contributions

All authors listed, have made substantial, direct and intellectual contribution to the work, and approved it for publication.

## Conflict of Interest Statement

The authors declare that the research was conducted in the absence of any commercial or financial relationships that could be construed as a potential conflict of interest.
